# Dr. John M. Johnson

**Published:** 1875-09

**Authors:** Jno. M. Johnson


*Editors Atlanta Medical and Surgical Journal:*

I am not vain enough to believe that my poor utterances are likely to mislead the profession, but I prefer if my opinions go to the public at all that they should present the real facts in the case. In the proceedings of the Academy of Medicine, appearing in the last number of the Journal, I am represented as saying that I would treat all fibroids with muriated tinct. of iron. I said exactly the opposite.

The question was a report of the removal of a uterine polypus by Dr. W. F. Westmoreland. I said I had not failed to remove them successfully by the use of muriated tincture of iron. Dr. W. asked if I proposed to treat all fibroids with this remedy. I said certainly; if all fibroids are alike, why vary the treatment? I then asked him if he still held to the opinion he expressed the day before, that polypi were fibroids, and all fibroids were alike; and he jocularly said yes. Of course this was mere tit for tat. I then said that while I had treated successfully mucous polypi in this way, I would treat fibroid tumors and growths by other methods.

Jno. M. Johnson, M.D.

